# Prevalence, Awareness, Treatment and Control of hypertension in the Malaysian population: findings from the National Health and Morbidity Survey 2006–2015

**DOI:** 10.1038/s41371-018-0082-x

**Published:** 2018-06-13

**Authors:** Nur Liana Ab Majid, Mohd Azahadi Omar, Yi Yi Khoo, Balkish Mahadir Naidu, Jane Ling Miaw Yn, Wan Shakira Rodzlan Hasani, Halizah Mat Rifin, Hamizatul Akmal Abd Hamid, Tania Gayle Robert Lourdes, Muhammad Fadhli Mohd Yusoff

**Affiliations:** 1 Institute for Public Health, Bangsar, Kuala Lumpur, Malaysia; 2 University of Malaya, Kuala Lumpur, Malaysia; 3 Department of Statistic Malaysia, Kuala Lumpur, Malaysia

## Abstract

Hypertension is strongly associated with chronic diseases such as myocardial infarction, stroke, heart failure, and renal failure. The objective of this study is to determine the trend of prevalence, awareness, treatment, and control of hypertension among Malaysian population since 2006 to 2015. The study used the data from National Health and Morbidity Survey (NHMS) 2006, 2011, and 2015. It was a cross-sectional with two-stage stratified random sampling throughout Malaysia for eligible respondents 18 years old and above. Respondents were interviewed face to face and blood pressure was recorded as the average reading from two electronic pressure monitoring measurements. Data was analyzed using the Complex sample module in SPSS Version 20. The prevalence of hypertension in Malaysia was 34.6% (95% CI: 33.9, 35.3) in 2006, 33.6% (95% CI: 32.6, 34.6) in 2011 and 35.3% (95% CI: 34.5, 36.3) in 2015. Awareness of hypertension in 2006, 2011, and 2015 was 35.6% (95% CI: 34.6, 36.6), 40.7% (95% CI: 39.3, 42.1), and 37.5% (95% CI: 36.1, 38.9) respectively. The trend of receiving treatment from 2006 to 2015 was 78.9% (95% CI: 77.5, 80.2) to 83.2% (95% CI: 81.3, 84.8). The control of hypertension increased significantly from 27.5% (95% CI: 25.9, 29.2) in 2006 to 37.4% (95% CI: 35.3, 39.5) in 2015. Despite higher proportions receiving treatment over time, the control of hypertension remained below 40% since NHMS 2006 until 2015. The strategies to further reduce the prevalence and increase awareness of hypertension should be enhanced particularly among the targeted age group to ensure early detection, treatment, and control thus preventing from long-term complications.

## Introduction

Hypertension is a global public health issue [1] strongly associated with chronic diseases such as myocardial infarction, stroke, heart failure, and renal failure. It is estimated that 6% of deaths worldwide are due to high blood pressure (BP) [2] but it is a preventable disease and a modifiable risk factor [3, 4]. Cardiovascular disease (CVD) is the most common cause of death in developed countries and a rapidly evolving cause of mortality and morbidity in economically developing countries [5]. In Malaysia, 36% of the mortality is due to CVD and, furthermore, one of the major cause of premature mortality [6, 7].

Like many other countries, CVD and hypertension have increased in Malaysia over time. Part of this is due to improved screening and diagnosis as medical services improve in the country. Early screening and detection of hypertension can reduce its complications, hence, its associated mortality rate. This will reduce the disease burden socially and economically.

Community surveillance has been the most common approach to evaluating the success of efforts to treat and control high BP. Although surveys are not a perfect evaluation tool, they are necessary to obtain information about persons who are, hitherto, unaware that they have hypertension or those who are aware but not compliant with medical advice [4].

In this regard, a nationwide community survey, the National Health and Morbidity Survey (NHMS), has been undertaken periodically in Malaysia since 1986 to assess the Nation’s health. These nationwide surveys have revealed a rising prevalence of hypertension in the country, similar to many other developing countries. In NHMS 1996, one-third of the population aged 30 years and above was diagnosed with hypertension. The survey also found very low awareness, treatment, and control among hypertensive respondents. Recently, the data from NHMS 2015 were made available, thus, we were able to compile the results for hypertension from 2006 until 2015 to look at the trend of hypertension in Malaysia. This trend will provide us with information regarding the change in hypertension prevalence over 10 years. Analysing the change will provide evidence on the direction of our healthcare services for the population.

## Methodology

All National Health and Morbidity Surveys (NHMS) were conducted by the Institute for Public Health under the Ministry of Health Malaysia. The Survey was conducted once every 10 years from 1986 to 2006, and thereafter, at 4-year intervals. In this study, we analyzed the data from NHMS 2006, 2011 and 2015. The study was approved by Medical Review & Ethics Committee within Ministry of Health. All respondents provided written informed consent.

The sampling design was cross-sectional with two-stage stratified random selection throughout Malaysia provided by Department of Statistics Malaysia. The first stage sampling unit was the Enumeration Block (EB) and, within each sampled Enumeration Block, the Living Quarters (LQ) were selected as the second stage. All households and eligible persons within each selected LQ were included in the study. Eligibility criteria depended on individual surveys or sub-survey. For the hypertension data, eligible respondents were 18 years and above. The estimated sample size at the national level was based on the stratification of respondents by states and by urban/rural.

Background data were obtained by face to face interview with each respondent after obtaining written informed consent. Blood pressure was recorded as the average reading from two electronic pressure monitoring measurements. The blood pressure measurement, weight and height were carried out by trained nurses.

All household members age 18 years and above were examined for their blood pressure. Two readings of systolic and diastolic pressure within 15 min apart were taken using Omron Digital Automatic Blood Pressure Monitor Model HEM-907 which was already validated and calibrated [8]. Blood pressure was taken using the standard procedure as below [9]:

- Without smoking, meal, caffeine intake, or physical exercise for at least 15 min
- Seated position, back supported, arm supported
- Seated with legs uncrossed, not talking, and relaxed
- The correct cuff bladder placed at heart level with the correct cuff size.

Blood pressure results were obtained and immediately recorded in the questionnaire. Respondents were informed of the results and, if found to be hypertensive (systolic blood pressure ≥140 mmHg or a diastolic blood pressure ≥90 mmHg), they were referred to the nearest health facility clinic for further evaluation and management.

Height was measured in centimeter using SECA Stadiometer 213 and weight was measured in kilogram using Tanita Personal Scale HD 319. Body mass index was calculated as weight in kilograms divided by height in meters squared and grouped into three categories according to WHO guidelines: <25.0 kg/m^2^ as underweight to normal weight, 25.0 to 29.9 kg/m^2^ as overweight and ≥30.0 kg/m^2^ as obesity.

### Definitions

Hypertension: respondents were classified as having hypertension if their blood pressure was ≥140 mmHg systolic or ≥90 mmHg diastolic or told to have hypertension by medical personnel previously. This protocol is in accordance with the Seventh Annual Report of the Joint National Committee [10].

Awareness of hypertension: awareness (yes/no) was defined as having ever been told to have hypertension by a medical doctor or paramedic among all hypertensive respondents.

Current treatment for hypertension: respondents taking antihypertensive medication within 2 weeks at the time of interview.

Control of hypertension: controlled hypertension was defined as having a desirable blood pressure level (<140/<90 mmHg).

### Statistical analysis

Data from the NHMS 2006, 2011, and 2015 were analyzed using the Complex sample module in SPSS Version 20 [11]. Sampling errors were estimated using the primary sampling units and strata provided in the data set. Sampling weights were used to adjust for non-response bias. As the sampling was two-stages stratified in design, the analysis was done accordingly to ensure sample weights and design effect was accounted for. Age adjusted values were standardized to the age distribution of the Malaysian population from the 2015 Census. The overall prevalence, as well as the awareness, treatment, and control rates were determined. A comparison of findings is possible as all NHMS surveys used a similar methodology.

## Results

The demographic characteristics of survey respondents are shown in Table 1. The total number of respondents aged 18 years old and above for the Blood pressure module was 72,573. The number of respondents in NHMS 2006, 2011, and 2015 was 34,539, 18,098, and 19,936 respectively. More than half of the respondents for each NHMS were Malays. For all consecutive NHMS, there were a higher number of female respondents, from urban areas and in the age group 18–39 years old. From 2006 to 2015, the percentage of overweight and obese respondent increased by 3.2% and 5.7% respectively, while respondents with BMI <25.0 kg/m^2^ decreased by 9.0%.

**Table 1:** Sociodemographic characteristic from National Health and Morbidity Survey 2006, 2011, and 2015

Overall	2006		2011		2015	
72,573	34,539		18,098		19,936	
Sex, *n* (%)	*n*	%	*n*	%	*n*	%
Male	15,458	44.8	8460	46.7	9483	47.6
Female	19,081	55.2	9638	53.3	10,453	52.4
Locality (%)						
Urban	20,492	59.3	10,543	58.3	11,505	57.7
Rural	14,047	40.7	7555	41.7	8431	42.3
Age group, *n* (%)						
18–39	16,565	48.0	8612	47.6	8906	44.7
40–59	13,020	37.7	6740	37.2	7240	36.3
60 above	4954	14.3	2746	15.2	3790	19.0
Ethnicity, *n* (%)						
Malay	18,983	55.0	10309	57.0	12,345	61.9
Chinese	7020	20.3	3495	19.3	3193	16.0
Indian	2845	8.2	1451	8.0	1410	7.1
Other Bumiputras	3954	11.4	1717	9.5	1751	8.8
Others	1737	5.0	1126	6.2	1237	6.2
BMI						
<25 kg/m^2^	18,835	57.1	8843	52.9	8903	48.1
25–29.9 kg/m^2^	9554	29.0	5130	30.7	5950	32.2
≥30 kg/m^2^	4608	14.0	2737	16.4	3646	19.7

*BMI* body mass index

### Prevalence of hypertension

The data were analyzed for all three NHMS, however, only the trend from 2006 to 2015 will be described. Details regarding prevalence, mean systolic and diastolic blood pressure, and awareness were listed in Table 2, age-adjusted to the Malaysian 2015 population. The prevalence of hypertension in Malaysia increased from 34.6% (95% CI: 33.9, 35.3) in 2006 to 35.3% (95% CI 34.5, 36.3) in 2015 however it was not statistically significant. The prevalence by sex has remained static for the past 10 years. In all three NHMS, the prevalence was higher among respondents from rural areas compared to urban, higher at age 60 years and above compared to younger age-groups and highest among obese respondent (BMI >30.0 kg/m^2^). By ethnic background, Chinese had the highest prevalence in 2006 but this decreased in trend in 2015. In fact, the overall mean SBP and DBP were significantly lower by 5.7 mmHg and 3.6 mmHg, respectively, in the 2015 survey compared to 2006.

**Table 2:** Prevalence and awareness of hypertension from National Health and Morbidity Survey 2006, 2011, and 2015

Variable	Prevalence of hypertension			Awareness among all hypertensives		
	2006	2011	2015	2006	2011	2015
	% (95% CI)	% (95% CI)	% (95% CI)	% (95% CI)	% (95% CI)	% (95% CI)
Overall	34.6 (33.9, 35.3)	33.6 (32.6, 34.6)	35.3 (34.5, 36.3)	35.6 (34.6, 36.6)	40.7 (39.3, 42.1)	37.5 (36.1, 38.9)
Sex						
Male	35.3 (34.4, 36.2)	34.2 (32.9, 35.4)	35.9 (34.7, 37.1)	31.1 (29.8, 32.4)	36.2 (34.3, 38.1)	33.8 (32.0, 35.7)
Female	33.9 (33.1, 34.7)	33.0 (31.7, 34.3)	34.8 (33.7, 36.0)	40.6 (39.3, 41.8)	45.6 (43.8, 47.5)	41.5 (39.7, 43.3)
Locality						
Urban	32.9 (32.1, 33.8)	32.6 (31.4, 33.9)	34.1 (33.0, 35.2)	37.2 (35.9, 38.4)	41.2 (39.4, 43.0)	38.6 (36.8, 40.3)
Rural	39.6 (38.6, 40.6)	36.5 (35.0, 38.1)	39.2 (37.6, 40.7)	31.6 (30.3, 32.9)	39.3 (37.4, 41.3)	34.6 (32.8, 36.5)
Age group						
18–39	17.6 (16.9, 18.3)	17.4 (16.4, 18.4)	18.9 (17.9, 19.9)	15.5 (14.2, 17.1)	22.2 (20.1, 24.6)	16.9 (14.9, 19.0)
40–59	48.8 (47.8, 49.8)	47.1 (45.7, 48.6)	49.5 (48.2, 50.9)	39.1 (37.7, 40.4)	43.2 (41.3, 45.2)	39.5 (37.7, 41.4)
60 above	73.1 (71.7, 74.5)	70.4 (68.4, 72.4)	71.9 (70.2, 73.5)	50.6 (48.9, 52.4)	56.1 (53.6, 58.5)	57.2 (55.0, 59.4)
BMI (kg/m^2^)						
<25	24.4 (23.7, 25.2)	22.8 (21.6, 23.9)	23.7 (22.6, 24.8)	28.3 (26.9, 29.7)	34.3 (32.2, 36.5)	32.3 (30.1, 34.5)
25–29	46.1 (45.0, 47.3)	42.2 (40.5, 43.9)	43.8 (42.4, 45.3)	38.0 (36.5, 39.6)	41.1 (38.9, 43.3)	38.9 (36.8, 41.0)
30 and above	57.8 (56.1, 59.4)	56.2 (54.1, 58.3)	57.1 (55.2, 58.9)	41.9 (39.9, 43.9)	43.6 (40.8, 46.4)	37.2 (34.8, 39.5)
Ethnicity						
Malay	35.4 (34.5, 36.3)	33.4 (32.1, 34.8)	36.4 (35.3, 37.5)	34.1 (32.9, 35.3)	39.8 (38.0, 41.6)	35.1 (33.4, 36.8)
Chinese	36.1 (34.7, 37.6)	35.9 (33.9, 38.0)	34.2 (32.3, 36.2)	40.2 (38.1, 42.4)	45.9 (42.9, 49.0)	46.1 (42.9, 49.3)
Indian	32.5 (30.6, 34.4)	34.3 (31.8, 37.0)	34.9 (32.0, 38.0)	41.2 (37.9, 44.7)	44.2 (39.1, 49.4)	46.3 (41.7, 51.1)
Other Bumiputras	33.3 (31.5, 35.2)	35.5 (32.8, 38.4)	37.3 (34.5, 40.2)	34.0 (31.3, 36.7)	37.0 (33.1, 41.2)	39.5 (35.2, 44.0)
Others	26.1 (23.7, 28.6)	24.0 (20.8, 27.6)	27.7 (24.9, 30.8)	19.4 (15.9, 23.4)	26.3 (20.5, 33.0)	21.5 (16.9, 27.0)
Mean SBP	130.66 (130.33, 131.00)	128.54 (128.03, 129.05)	124.93 (124.11, 125.75)			
Mean DBP	79.83 (79.65, 80.02)	79.59 (79.32, 79.86)	76.27 (75.76, 76.77)			

^a^Age adjusted to population in the year 2015

### Awareness, treatment, and control of hypertension

Compared to 2006, respondents’ awareness of hypertension was higher in 2015 in every age group, and by gender, locality, and ethnicity. Higher awareness was seen among females compared to males, Chinese and Indian respondents compared to Malays, respondents living in urban compared to rural areas, and respondents age 60 years old and above. A higher proportion of respondents with BMI <25.0 kg/m^2^ were aware in 2015 than in 2006. At the same time, however, awareness among obese respondents was significantly lowered by 4.7%.

Details regarding treatment and control are listed in Table 3. Overall, a significantly higher (by 3%) proportion of hypertensive respondents were receiving treatment in 2015. The trend of receiving treatment was highest among female, Chinese and Indian, respondents in urban, respondents age 60 years old and above and normal/obese respondents. The proportion receiving treatment was also higher among those who were aware of having hypertension by 4.3% since 2006. Similar trends were seen among all hypertensives and among those aware of having hypertension.

**Table 3:** Treatment and control of hypertension from National Health and Morbidity Survey 2006, 2011, and 2015

Variable	Treatment among all hypertensives			Treatment among those aware			Control among treated		
	2006	2011	2015	2006	2011	2015	2006	2011	2015
	% (95% CI)	% (95% CI)	% (95% CI)	% (95% CI)	% (95% CI)	% (95% CI)	% (95% CI)	% (95% CI)	% (95% CI)
Overall	28.1 (27.2, 29.0)	30.6 (29.3, 32.0)	31.1 (29.8, 32.5)	78.9 (77.5, 80.2)	77.5 (75.5, 79.4)	83.2 (81.3, 84.8)	27.5 (25.9, 29.2)	34.3 (32.0, 36.7)	37.4 (35.3, 39.5)
Sex									
Male	23.7 (22.5, 24.9)	26.9 (25.1, 28.6)	27.5 (25.9, 29.3)	76.2 (74.0, 78.3)	76.4 (73.4, 79.2)	81.6 (78.9, 84.0)	29.5 (27.0, 32.2)	35.8 (32.3, 39.5)	39.3 (36.2, 42.4)
Female	32.9 (31.7, 34.1)	34.8 (33.0, 36.7)	35.1 (33.4, 36.8)	81.1 (79.4, 82.6)	78.5 (76.1, 80.8)	84.6 (82.3, 86.6)	25.9 (24.0, 27.9)	33.1 (30.3, 36.0)	35.7 (33.0, 38.6)
Locality									
Urban	29.9 (28.8, 31.1)	31.3(29.6, 33.1)	32.1 (30.4, 33.8)	80.5 (78.8, 82.2)	78.2 (75.7, 80.6)	83.2 (80.9, 85.3)	29.3 (27.4, 31.3)	36.0 (33.1, 39.0)	38.7 (36.1, 41.4)
Rural	23.3 (22.1, 24.5)	28.8(27.1, 30.7)	28.7 (27.0, 30.4)	73.8 (71.6, 75.9)	75.5 (72.6, 78.2)	83.0 (80.5, 85.3)	21.7 (19.5, 24.1)	29.5 (26.5, 32.8)	33.3 (30.6, 36.1)
Age group									
18–39	7.5 (6.5, 8.6)	8.7 (7.3, 10.3)	8.4 (7.0, 9.9)	48.2 (43.2, 53.2)	41.9 (36.3, 47.7)	49.6 (43.6, 55.7)	32.6 (26.4, 39.5)	35.4 (27.4, 44.5)	39.4 (31.4, 48.0)
40–59	31.1 (29.8, 32.4)	33.4 (31.6, 35.3)	33.3 (31.5, 35.1)	79.6 (77.9, 81.3)	79.2 (76.6, 81.5)	84.3 (82.0, 86.4)	29.6 (27.3, 31.9)	33.0 (29.9, 36.3)	36.7 (33.7, 39.9)
60 above	44.4 (42.6, 46.2)	49.3 (46.8, 51.8)	53.1 (50.9, 55.4)	87.6 (85.9, 89.1)	89.7 (87.4, 91.7)	92.9 (91.3, 94.2)	24.3 (22.0, 26.8)	35.5 (32.0, 39.2)	37.7 (34.9, 40.6)
BMI									
<25	22.4 (21.1, 23.7)	26.0 (24.0, 28.1)	26.5 (24.5, 28.5)	79.1 (76.8, 81.3)	78.0 (74.6, 81.1)	82.1 (78.6, 85.1)	31.0 (28.1, 34.0)	40.8 (36.5, 45.2)	43.3 (39.3, 47.4)
25–29	29.6 (28.2, 31.1)	31.0 (28.9, 33.2)	32.2 (30.2, 34.2)	77.9 (75.7, 79.9)	77.5 (74.1, 80.6)	82.9 (80.3, 85.2)	24.7 (22.3, 27.3)	33.8 (30.2, 37.5)	31.3 (28.3, 34.4)
30 and above	33.4 (31.5, 35.3)	31.6 (29.1, 34.2)	30.9 (28.7, 33.3)	79.7 (77.0, 82.1)	74.6 (70.7, 78.1)	83.3 (79.8, 86.2)	26.9 (24.0, 30.0)	27.0 (23.3, 31.1)	32.4 (28.6, 36.4)
Ethnicity									
Malay	25.9 (24.7, 27.0)	28.6 (26.9, 30.3)	29.3 (27.7, 31.0)	75.8 (73.8, 77.7)	73.7 (70.8, 76.3)	83.7 (81.4, 85.7)	24.4 (22.3, 26.7)	29.8 (26.9, 33.0)	33.2 (30.7, 35.9)
Chinese	35.4 (33.3, 37.6)	38.7 (35.7, 41.8)	40.6 (37.3, 43.9)	88.1 (85.9, 90.0)	87.4 (84.2, 90.1)	88.1 (84.3, 91.0)	31.6 (28.4, 34.9)	42.5 (37.8, 47.4)	46.8 (41.9, 51.8)
Indian	34.7 (31.6, 37.9)	36.9 (31.8, 42.3)	41.3 (36.9, 45.9)	84.1 (79.7, 87.6)	84.2 (78.2, 88.8)	89.5 (84.5, 93.0)	33.7 (28.6, 39.3)	40.4 (34.2, 46.8)	40.8 (33.9, 48.1)
Other Bumiputras	22.6 (20.3, 25.1)	24.6 (21.0, 28.7)	26.6 (22.8, 30.8)	66.6 (61.6, 71.3)	69.7 (62.9, 75.7)	67.3 (60.0, 73.9)	25.5 (20.9, 30.6)	31.4 (24.3, 39.4)	42.2 (35.1, 49.5)
Others	11.7 (9.0, 15.1)	15.3 (10.7, 21.3)	13.9 (10.4, 18.2)	60.4 (49.8, 70.2)	61.6 (48.3, 73.3)	64.4 (52.3, 74.9)	14.2 (7.2, 26.0)	15.6 (7.9, 28.5)	20.2 (11.4, 33.2)

^a^Age adjusted to population in the year 2015

From 2006 to 2015, male had higher control compared to female but it was not statistically significant. Chinese and other Bumiputras had the highest proportion and increment for blood pressure control. Similar trends were seen for the locality, the proportion was higher among urban and among those with lowest BMI category. The highest proportion for blood pressure control was among age group 18–39 years old, however, the increment of the BP control was highest among the age group 60 years old and above (13.4%). Despite higher proportions receiving treatment over time, the control of hypertension remained below 40% among those who received treatment since NHMS 2006 until 2015.

## Discussion

The prevalence of hypertension in Malaysia from the first nationwide sampled survey of adults aged 18 years and above in 2006 to the one in 2015 appears to be stable with minimal increases. Be that as it may, a rise of 0.7% from 2006 to 2015 nationwide can be extrapolated to be around 184,000 Malaysians. The prevalence of hypertension in Malaysia remains above 30% and is higher than other countries, such as China and Singapore [4]. The trend of prevalence in Malaysia is more similar to developed country rather than developing countries [12]. Despite increasing hypertension prevalence, the SBP and DBP showed reduction since 2006 and is consistent with other studies [13].

In this report, we only analyzed the data from NHMS 2006, 2011, and 2015. Data on Malaysians 30 years old and above are available from NHMS carried out in 1996. Confining data for respondents aged 30 and above for all surveys, the prevalence of hypertension in Malaysia was 32.9% in 1996 [14] and Rampal et al. reported hypertension prevalence of 40.5% in 2004 [15]. For NHMS 2006, 2011, and 2015 the prevalence was 44.6, 43.8, and 44.7% respectively (data not shown). The trend showed that the prevalence of hypertension has increased ~11.8% in 20 years duration. The reason behind the increase in trend for the prevalence was not specifically mentioned anywhere however with the trend of increasing of obesity in Malaysia [16] this could be one of the causes contributing to the increase of the prevalence of hypertension [17]. According to Guo et al., there were other contributing factors for this high prevalence such as increasing consumption of dietary sodium, the increasingly sedentary lifestyle and the suboptimal levels of health literacy among the community [17].

Unlike many other countries such as Saudi Arabia, Portugal, and China [18–20] where prevalence is higher among male than female, there was no significant difference by gender in Malaysia. Awareness of hypertension, on the other hand, was higher in females compared with males, a finding similar to many international studies like Thailand and India [21, 22]. Among all hypertensive respondents, the females were more likely to receive treatment. However, the finding from NHMS 2006–2015 showed that males have a better blood pressure control compared to females but it was not statistically significant. These findings were in contrary to other studies and according to Wolf-Maier et al., females always better treated because they were considered as lower CVD risk patients [2].

The prevalence of hypertension was consistently higher among the rural respondents since 2006 compared to the respondents in urban possibly due to urbanization of the lifestyle factor from urban to rural as described by Abdul Razak et al. [23]. However, this is contrary to other studies, e.g., in India [24] and China [25], where a higher prevalence was reported for urban respondents. This is most likely due to the migration of the younger population from rural to urban area [26]. Respondents from urban had higher awareness regarding their hypertension status, received treatment and had their blood pressure under control than respondents in rural areas which according to Wang et al., was due to higher socioeconomic status [20]

The prevalence of hypertension in our study increased with age, another finding supporting previous studies in other countries, such as Saudi Arabia [18], Palestine [27], and Greece [28]. This is not unexpected since hypertension is a consequence of the degenerative aging process resulting in the thickening and loss of elasticity in arteries [27]. Within each survey, awareness and treatment also increased with age, but very minimally from age group for respondents under 60 years old. Based on NHMS 2006 data, Ho et al. had described the same finding in relation to awareness and age. They postulated that the elderly may be more health conscious and have better access to medical care and treatment [29]. The control of hypertension increased significantly from 2006 to 2015 among respondents age 40 years and above and according to Benegas et al., the control among elderly is higher most likely due to higher treatment rates and more intense drug treatment [30].

The prevalence of hypertension increases among all ethnicity except Chinese, this possibly could be due to genetic factor according to R Gupta et al. or differences in traditional Chinese diet of vegetables and grains [26] but whether Chinese Malaysians have a similar diet is still unknown. The awareness increased among all ethnicity with highest among the Chinese and Other Bumiputras from 2006 to 2015.

The treatment was higher in 2015 compared to 2006 among all ethnicities. However, the highest increment was among Malays. The control was increasing among all ethnicities with the highest increment among the Other Bumiputras.

Obesity is one of the most common risk factors for hypertension [31]. From NHMS 2006 to 2015, the prevalence of hypertension increased with higher body mass index. However, the trend of awareness was reduced in the obese group whereas it increased among those with lower BMI. The treatment and control increased among all BMI categories from 2006 to 2015. Not much difference in the increment of the treatment among all BMI categories but the highest increment of control was among lower BMI category. This could be due to other multi-factorial causes such as level of physical activity, high salt intake or low potassium intake that appeared to be the major contributors to hypertension [32].

The prevalence of treatment among respondents with hypertension in Malaysia was similar to other countries like in the England, Spain and Germany [2]. The prevalence was higher among those who were aware of their hypertensive status and this could be due to the continuous and intensive effort by the Ministry of Health Malaysia to combat NCD through community program. The programs enhanced the screening, early referrals and integration of management in primary care as mentioned by Mustapha et al. [33]. In addition, the Malaysian government also provided medical treatment and services at a very affordable price where patient was only required to pay RM1(~0.25 USD) for outpatient care [34]. Among those who received treatment for hypertension, many still have poor blood pressure control. This could be due to poor adherence to medication as reported by Azuana et al. [35].

### Public health implication

The prevalence of hypertension in Malaysia has shown an increase of 0.7% over the last decade. The high prevalence of hypertension among the elderly age group is noted to be the main contributor to the national prevalence. Meanwhile, the youngest age group have shown the lowest awareness since 1996. Three-quarters of the hypertensive population sought treatment and only <50% of them had their blood pressures under control.

In order to tackle the problem of hypertension in Malaysia, the Ministry of Health has taken the lead in formulating the National Strategic Plan for Non-Communicable Diseases 2016–2025 (NSP-NCD 2016–2025), following up from the earlier NSP-NCD 2010–2014, to combat NCDs where the risk factors are already known and well described.

NSPNCD 2016–2025 was an initiative from the Eleventh Malaysia Plan (2016–2020). There are seven action plans and initiatives within the NSPNCD which include Tobacco Control, Obesity Control, Salt Reduction Strategy, Active Living, Alcohol Control, Cancer Control and Strengthening Chronic Disease Management at Primary Care Level through the Enhanced Primary Health Care (EnPHC) Initiative [36].

The strategies of NSPNCD should be enhanced to further reduce the prevalence of hypertension particularly among the elderly. Simultaneously, awareness and screening of hypertension should be increased among the younger age group to ensure early detection and treatment thus preventing long-term complications. For better control of hypertension, regular monitoring and supervision are needed among all medical practitioners to be in-line with the Clinical Practice Guideline to make sure all patients achieve their target blood pressure. As Malaysia is a multi-racial and multicultural country, further studies are needed to explore other possible causes of hypertension such as genetic factors, environmental and socio-cultural influence.

### Limitations

There are several limitations in this study. This must be taken into consideration because the BP measurement was taken during a single visit [37] which possibly resulted in the overestimation in the prevalence of hypertension and underestimation of control of elevated BP. According to the Malaysian Clinical Practice Guidelines (CPG), the diagnosis of hypertension is defined as persistent elevation of systolic BP of 140 mmHg or greater and/or diastolic BP of 90 mmHg or greater based on the average of two or more properly measured seated, BP readings on each of two or more visits [9].The limitation is similar to many other international studies and the limitations of the study design for NHMS 2006, 2011 and 2015 were the same. Despite its limitation, the trend did provide an important overview of hypertension in Malaysia for 10 years.

### Study Highlights

#### What is known about this topic

- Globally, prevalence of hypertension is increasing in trend.
- Many countries had conducted national survey to determine the prevalence, awareness, treatment, and control of hypertension in their country.

#### What this study adds

- Trends of hypertension in Malaysia can be analyzed from multiple National Health and Morbidity survey that were conducted.
- National Health and Morbidity Survey 2006, 2011, and 2015 in Malaysia will provide the trends of prevalence, awareness, treatment, and control of hypertension for 10 years.
